# Current Insights and Future Prospects for Targeting IL-17 to Treat Patients With Systemic Lupus Erythematosus

**DOI:** 10.3389/fimmu.2020.624971

**Published:** 2021-02-01

**Authors:** Tomohiro Koga, Kunihiro Ichinose, Atsushi Kawakami, George C. Tsokos

**Affiliations:** ^1^ Division of Advanced Preventive Medical Sciences, Department of Immunology and Rheumatology, Nagasaki University Graduate School of Biomedical Sciences, Nagasaki, Japan; ^2^ Center for Bioinformatics and Molecular Medicine, Nagasaki University Graduate School of Biomedical Sciences, Nagasaki, Japan; ^3^ Division of Rheumatology and Clinical Immunology, Department of Medicine, Beth Israel Deaconess Medical Center, Harvard Medical School, Boston, MA, United States

**Keywords:** T cells, systemic lupus erythematosus (SLE), lupus nephritis, immune responses, interleukin (IL)-17

## Abstract

Systemic lupus erythematosus (SLE) is an autoimmune disease characterized by immune cell abnormalities which lead to the production of autoantibodies and the deposition of immune complexes. Interleukin (IL)-17-producing cells play an important role in the pathogenesis of the disease, making them an attractive therapeutic target. Studies in lupus-prone mice and of *ex vivo* cells from patients with SLE humans have shown that IL-17 represents a promising therapeutic target. Here we review molecular mechanisms involved in IL-17 production and Th17 cell differentiation and function and an update on the role of IL-17 in autoimmune diseases and the expected usefulness for targeting IL-17 therapeutically.

## Introduction

Systemic lupus erythematosus (SLE) is a heterogeneous autoimmune disease characterized by the production of autoantibodies, the formation of immune complexes, and immune dysregulation, resulting in damage of multiple organs, including the skin and the kidneys (1, 2). The prognosis for SLE depends on the severity of the disease and the organs that are involved. Lupus nephritis (LN) is the most common and serious complication observed in the majority of patients with SLE. While the etiology of SLE remains largely unknown, genome-wide association studies have identified over 50 gene loci with variants that have been associated with a predisposition to SLE (3–5). These disease-susceptible genes for SLE include variants that have been implicated in aberrant expression of cytokines and abnormalities in innate and adaptive immunity.

Both B cells and T cells are important in the pathogenesis of SLE. Self-reactive B cells that produce autoantibodies are important in the pathogenesis of SLE. Increased plasma memory B cell subsets are associated with disease activity, and therapies targeting B cells have shown some clinical improvement (6). T cells also play a central role in the production of autoantibodies and the subsequent formation of immune complexes. Both B and T cells may act in concert to induce direct damage in multiple organs (7, 8).

CD4+ T helper cells (Th cells) are particularly important in the series of autoimmune responses associated with SLE. Th cells are defined by the cytokines they produce and have been classified into Th1, Th2, Th17, follicular helper T (Tfh) cells, and regulatory T (Treg) cells (7–9).

Th1 cells produce primarily interferon (IFN)-γ, which in turn activates cytotoxic T lymphocytes, macrophages, and natural killer cells. In contrast, Th2 cells produce mainly cytokines, such as interleukin (IL)-4 and activate B cells. The pathogenesis of autoimmune diseases, however, cannot be based on Th1 and Th2 immune responses alone. Th17 cells and Treg cells play important roles in the development of autoimmune-mediated tissue injury.

Th17 cells produce IL-17, IL-21, and IL-22, and they have been shown to be involved in the development of inflammation in various organs. Treg cells are characterized by the expression of FoxP3 and they produce TGF-β and IL-10, which actively terminate immune responses. Interestingly, there is an interrelationship between Th17 and Treg cells that may determine the ultimate outcome of the autoimmune response. Limited numbers and reduced functions of Treg cells have been observed in patients with SLE, and these defects have been associated with increased disease activity (3).

In this review, we discuss the evidence that T cell dysfunctions and IL-17 overproduction are associated with the development of SLE and disease progression in both humans and lupus-prone mice. We also describe recent advances in functional analysis, including analysis of the cell signaling pathways that contribute to increased IL-17 production. It is well understood that the imbalance between Th17 cells and Treg cells, along with IL-17-related cytokine-driven inflammation, plays an important role in autoantibody production and organ damage in SLE. We will also discuss recent advances in IL-17-targeted therapies for autoimmune diseases, including SLE, and their future prospects.

## The Role of Interleukin-17 and Interleukin-17-Related Cytokines in the Pathogenesis of Systemic Lupus Erythematosus

The IL-17 family includes at least six (IL-17A, IL-17B, IL-17C, IL-17D, IL-17E, and IL-17F) proteins (9). Among these, IL-17A, which is mainly produced by Th17 cells, amplifies the production of inflammatory cytokines and chemokines and stimulates keratinocytes, synoviocytes, fibroblasts, macrophages, and neutrophils (10). Accordingly, it has the potential to promote the recruitment of inflammatory cells, such as monocytes and neutrophils, to the inflamed organ (11, 12). Although Th17 cells produce mainly the cytokine IL-17 (12), IL-17 is also produced by other subsets of T cells, including T cell receptor (TCR)γδ and TCRαβ double negative (DN) T cells (CD3^+^CD4^−^CD8^−^), and a number of families of innate lymphoid cells, including ILC3, macrophages, and neutrophils (13–15).

IL-17A is an important cytokine that is involved in the pathogenesis of animal models of autoimmunity and human autoimmune diseases, including SLE (12, 16, 17). It has been demonstrated that patients with SLE not only have higher serum levels of IL-17A, but also have increased numbers of Th17 cells (18–20). It has also been shown that high serum levels of IL-17 at baseline predict poor histopathological outcomes after immunosuppressive therapy (21). Our group has proposed that DN T cells infiltrate the kidneys of patients with LN and are the major source of IL-17 (13). However, a study using lupus-prone mice demonstrated that pharmacological inhibition and genetic ablation of IL-17A did not improve clinical manifestations, including survival rate, glomerulonephritis, and autoantibody production (22). As mentioned above, Th17 cells not only produce IL-17, but also produce multiple pro-inflammatory cytokines, such as IL-21, IL-22, and TNF-α. Thus, the role of IL-17 that has been documented in other studies may not be due to IL-17 alone, but to the additional activity of Th17 cells. Therefore, studies in which Th17 cells, rather than IL-17 production alone, are involved in the pathogenesis of SLE need to formally address this issue.

IL-23 promotes signal transducer and transcriptional activator 3 (STAT3) phosphorylation by Janus kinase 2 (JAK2) and tyrosine kinase 2 (TYK2) by binding to its receptor IL-23R. It also enhances the expression of retinoic acid receptor-associated orphan receptor γt (RORγt), which is involved in the expression of IL-17 and other Th17 cytokines (23). Thus, IL-23 has been shown to be important in the development of various autoimmune diseases in murine models (24–26) and in humans (27) by promoting Th17 cell–mediated tissue inflammation. Our group has shown that the clinical and pathological findings of LN are mitigated in lupus-prone mice with IL-23 receptor deficiency (28) or treated with anti-IL23 antibodies (29). Evidence for the importance of IL-23 in SLE is further supported by the elevated serum IL-23 and IL-23 expression in renal tissues in patients with SLE (21, 30, 31).

Low-density granulocytes, a subpopulation of neutrophils prone to cell death, have been found to contribute to the pathogenesis of SLE through the neutrophil extracellular trap formation (NETosis) process, which includes the release of intracellular material into the surrounding environment (32, 33). IL-17 plays a role in inducing the recruitment of neutrophils and other immune cells by targeting tissues to promote and maintain the inflammatory process. In addition, IL-17 has been demonstrated to induce NETosis in animals prone to lupus (34). Indeed, the cellular debris released by these cells induces activation of the type I IFN pathway by plasmacytoid dendritic cells, which eventually leads to the aberrant activation of T and B cells (35, 36). This consequently perpetuates the inflammatory process characteristic of SLE.

## Molecular Mechanisms that Regulate Interleukin-17 in Systemic Lupus Erythematosus

CD4^+^ T cell dysfunction contributes to the development and progression of organ damage, including LN in lupus-prone mice, such as MRL/lpr, NZB/NZW, and BXSB mice, and SLE patients (37, 38). Molecules involved in the aberrant expression of IL-17 cytokines and distortion of Th cell differentiation include protein phosphatase 2A (PP2A), calcium/calmodulin kinase IV (CaMK4), CREM, Rho-associated protein kinase (ROCK), and mammalian target rapamycin complex 1 (mTORC1).

### Protein Phosphatase 2A

PP2A is a multifunctional serine/threonine phosphatase that is involved in multiple cellular processes. It is composed of three distinct subunits: the scaffold A subunit (PP2Aa), the regulatory B subunit (PP2Ab), and the catalytic C subunit (PP2Ac).

A previous study carried out by our group showed that transgenic mice that overexpressed PP2Ac in T cells developed glomerulonephritis, which included increased production of IL-17A and IL-17F (39). Consistent with these observations, it has been demonstrated that PP2Ac expression and activity are increased in the T cells of SLE patients, which contributes to a decrease in IL-2 production (40, 41). In addition, Treg cell-specific ablation of the PP2A causes multi-organ lymphoproliferative autoimmune diseases due to defective dephosphorylation of mTORC1 (42).

Recently, our group has shown that PPP2R2D, a regulatory subunit of PP2A, is increased in T cells from SLE patients. Mice lacking this subunit in T cells have less autoimmunity and PPP2R2D negatively regulates IL-2 production in conventional T cells by regulating the chromatin opening of the *IL-2* gene (43).

PP2A is a ubiquitously expressed enzyme. It also has diverse effects on immune cells. Therefore, the use of PP2A inhibitors to treat patients with SLE requires the use of a T cell-targeted delivery system to mitigate off-target effects.

### Rho-Associated Protein Kinase

ROCK is a serine-threonine kinase and its activity is primarily controlled by the binding of activated RhoA (44). ROCK is involved in regulating cell migration, including that of T cells (45). ROCK2 has been suggested to facilitate the activity of interferon regulatory factor 4 (IRF4), which is required for Th17 differentiation and the production of IL-17 and IL-21 (46). PP2Ac in T cells has also been shown to be involved in IL-17 production *via* promotion of the RhoA-ROCK-IRF4 pathway (47). A study showed that ROCK activity levels were significantly higher in SLE patients than in healthy controls and the inhibition of the RhoA-ROCK pathway suppressed the production of IL-17 and IL-21 by Th17 cells (48).

ROCK2 has also been shown to be a major ROCK isoform that is involved in the differentiation of Th17 cells generated under Th17 cell skewing conditions. Therefore, targeting of this pathway can be achieved by both selective and non-selective inhibitors. A better understanding of the functional relevance of the ROCK1-dependent pathway in immune cells and an evaluation of the pattern of ROCK expression in individual SLE patients is required to determine whether ROCK2-selective inhibitors provide a more favorable risk-benefit profile than the broader ROCK inhibitors.

### CREM

CREM is a member of the ATF/CREB-type bZip transcription factor family. It binds to cAMP response elements during cellular processes, including T cell activation. Therefore, CREM plays an important role in the adaptive immune process. Importantly, CREMα functions as a transcriptional regulator of molecules associated with cytokine expression and T cell differentiation in T cells of SLE patients.

Previous studies have demonstrated that mice overexpressing CREMα in T cells have increased IL-17 production and lupus-like disease (49). Mechanistically, CREMα was found to bind to the *IL17* promoter and non-coding conserved areas of the *IL17* locus and enhance its activity at the epigenetic level (50, 51). Consistent with the results obtained in mice, T cells from SLE patients have been found to have increased levels of CREMα and aberrant IL-17A expression (51). In addition, CREMα was found to be essential for expansion of DN T cells due to epigenetic regulation of the *CD8* locus cells in SLE patients and lupus-prone mice (52, 53). In summary, reduced levels of CREMα can suppress the production of IL-17 and reduce the pool of pathogenic DN T cells, which suggests its potential as a disease biomarker and therapeutic target in SLE.

The splice variant of CREM inducible cAMP early repressor (ICER) also has a crucial role in T cell activation, Th cell differentiation, and cytokine production (54). Experiments involving mice have demonstrated that ICER/CREM is required for the development of organ-specific autoimmunity and systemic autoimmunity and ICER is upregulated in CD4+ T cells from SLE patients (55).

Therefore, CREM and CREM-associated molecules may represent potential therapeutic targets for SLE. However, as with PP2A, the CREM family of proteins has an enormous diversity and the development of small molecule compounds that target only specific subunits or splice variants may pose many challenges.

### Calcium/Calmodulin Kinase IV

Calcium/calmodulin-dependent protein kinases (CaMKs) are enzymes that are activated by calcium. CaMK2 and CaMK4, which are multifunctional CaMKs with multiple substrates, play important roles in the immune response, including T cell activation (56, 57) and T cell development (58, 59). CaMK4 is a multifunctional serine/threonine kinase that regulates pro-inflammatory cytokines and cell proliferation-related gene expression by activating a number of transcription factors, including CREB (cAMP response element binding protein) and CREM (60).

CaMK4 is abnormally increased in T cells from SLE patients (61) and lupus-prone mice (62). CaMK4 is rarely expressed in B cells or other immune cells. Among T cells, CaMK4 expression is enhanced in CD4-positive T cells, and it is preferentially induced during Th17 differentiation (63). In line with these findings, genetic or pharmacological inhibition of CaMK4 in MRL/lpr mice resulted in a reduced frequency of IL-17-producing T cells, including CD4+ and DN T cells, a significant reduction in autoantibody production, and improved nephritis (62, 64). As a mechanism to counteract organ damage, we demonstrated that CaMK4 inhibition limits cell infiltration by increasing Treg cells locally in the kidney (65). Inhibition of CaMK4 suppress the CCR6/CCL20 axis which is important for the entry of Th17 cells to tissues (66). Moreover, we recently discovered that GLUT1-mediated glycolysis is important for the expression of IL-17 induced by CaMK4 (67).

Thus, targeting CaMK4 represents a potential therapeutic strategy for patients with SLE because of its ability to promote differentiation into Th17 cells. However, since CaMK4 is also upregulated in critical organs such as the brain and gonads, research on CD4-targeted therapy using nanolipogels (68), development of CaMK4-specific inhibitors, and verification of their safety must be conducted before further clinical applications are carried out in humans.

### Mammalian Target Rapamycin Complex 1

mTORC1 is a serine-threonine kinase that functions as a regulator of cellular metabolism, including mitochondrial oxidative stress, glycolysis (69), and cell proliferation (70). Rapamycin, an mTORC1 inhibitor, suppressed glomerulonephritis in lupus-prone mice (71) by suppressing the Th17/Treg cell ratio (72). Its molecular signaling mechanism has been suggested to be linked to CaMK4 (63), ROCK (73), and the splicing factor SRSF1 (74). Furthermore, recent studies have shown that rapamycin reverses Th17 cell proliferation in SLE patients (75, 76). Importantly, glutaminolysis has been shown to be essential for mTORC activation, and Th17 cells are more dependent on glutaminolysis than Th1, Th2, and Treg cells (77). Glutaminase 1 inhibitors improve disease activity and there are fewer IL‐17A–producing T cells in the kidneys of MRL/*lpr* mice (78).

Activation of the mTOR pathway is important in the development of SLE (79, 80). This allows mTOR to be a therapeutic target for SLE. A single-arm, open-label, phase 1 and 2 study of the mTOR inhibitor sirolimus showed efficacy in patients with active SLE (81). In summary, TORC1 inhibition has also shown several clinical benefits in patients with SLE.

**Figure 1:**
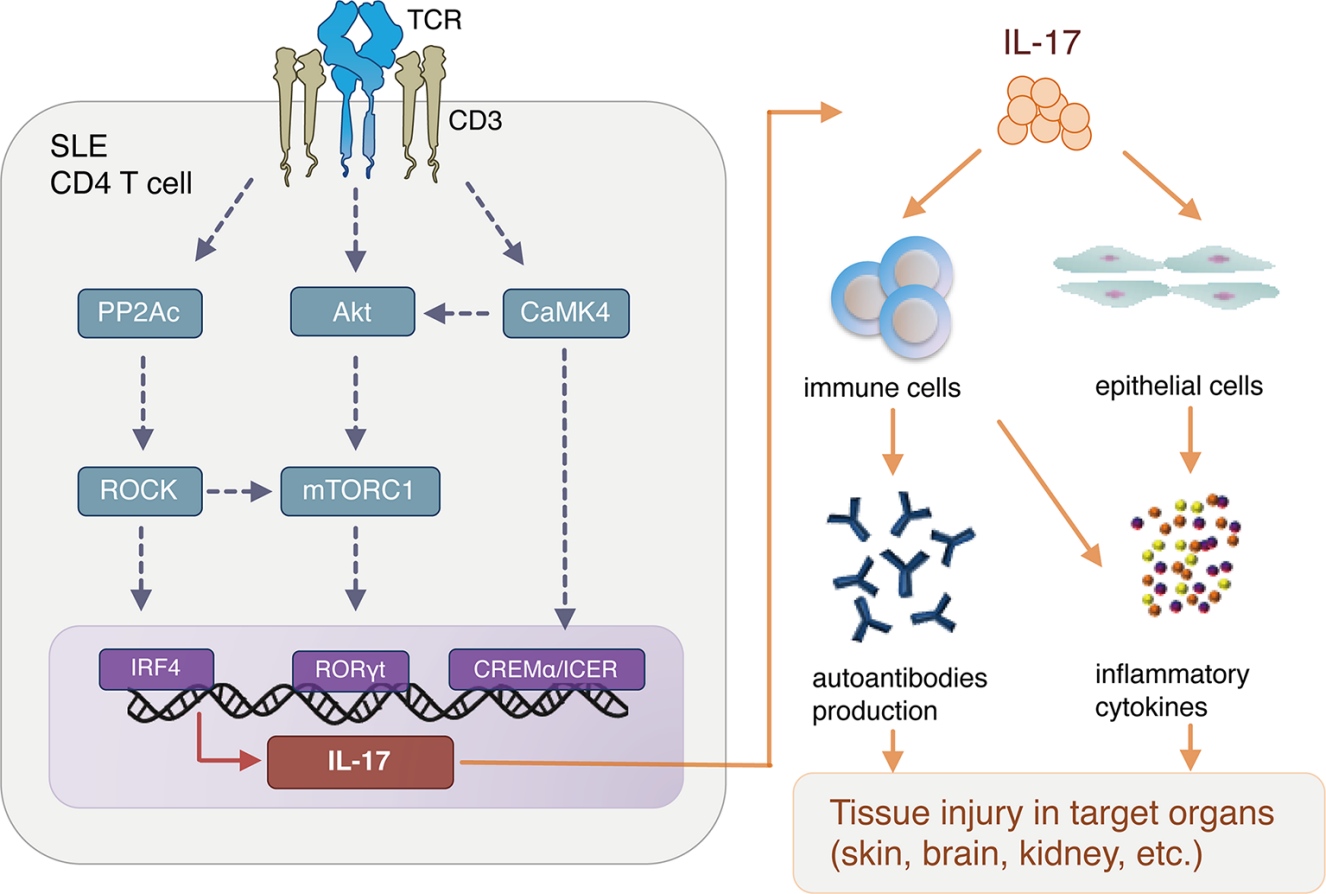
Aberrant T cell signaling and interleukin (IL)-17 production in SLE pathogenesis. SLE, systemic lupus erythematosus; TCR, T cell receptor; PP2Ac, Protein phosphatase 2A catalytic subunit; CaMK4, Calcium/calmodulin-dependent protein kinase IV; ROCK, Rho-associated protein kinase; CREB, cAMP response element binding protein; IRF4, interferon regulatory factor 4; CREM, cAMP response element modulator; mTORC1, mammalian target of rapamycin complex 1.

**Figure 1** summarizes evidence that abnormal T-cell signaling leads to overproduction of IL-17 in SLE, which in turn activates immune and other cells, leading to autoantibody production and proinflammatory cytokine production, resulting in organ damage.

## Targeting Interleukin-17 Therapy in Patients With Systemic Lupus Erythematosus

Several IL-17A blockers, including the anti-IL-17A monoclonal antibodies secukinumab, ixekizumab, and bimekizumab, and the anti-17RA monoclonal antibody brodalumab, are approved for some immune-mediated inflammatory diseases, such as psoriasis (82–84), psoriatic arthritis (85, 86), and ankylosing spondylitis (87, 88). Although a case report described the efficacy of IL-17A inhibitor in a SLE patient (89), clinical trials are warranted to evaluate the long-term efficacy and safety of IL-17 inhibitors in SLE patients.

Several issues should be considered in the development of IL-17-directed therapy for SLE. First, IL-17A blockers are already used in clinical practice for inflammatory diseases, but their long-term safety and efficacy have not been established. Second, anti-IL-17A drugs have been shown to be therapeutically effective in lupus-prone mice, but human studies are needed to determine the exact role of IL-17 in human SLE. Finally, because SLE is a highly heterogeneous autoimmune disease, IL-17 blockade may not be suitable for all patients. The potential beneficial effects of IL-17 blockers may be limited to a subset of SLE patients whose disease is driven by the IL-17 pathway. Therefore, it is important to identify biomarkers that can be used in patient screening to identify those who have the best chance to respond to treatment with IL-17 pathway-directed biologics.

Clinical studies have demonstrated the efficacy and safety of ustekinumab, an anti- IL-12/23 p40 neutralizing monoclonal antibody, in patients with subacute cutaneous lupus (90), psoriasis (91), and psoriatic arthritis (92). More recently, a double-blind phase II study has demonstrated impressive efficacy and safety of ustekinumab when used in patients with active SLE (93). An ancillary study to this trial revealed that persistent reductions in IFN-γ serum protein levels, rather than changes in serum IL-17A, IL-17F, and IL-22 levels, were associated with treatment responses (94).

## Conclusion and Future Perspectives

In this review, we report recent advances in our understanding of the role of IL-17 and IL-17-related molecules in SLE and their clinical implications. There is sufficient evidence that Th17 and one of their main effector molecules, IL-17, contribute to the development of immunopathology in patients and mice with lupus.

It is true that many biologics have been tried in patients with SLE and the vast majority of them have failed to produce a statistically significant effect admissible by the regulatory agencies even if phase II studies had indicated high promise. Obviously, each biologic accomplishes the expected biologic effect, that is, to neutralize a cytokine or kill a cell, and therefore, the blame should be directed to the design of the clinical trials.

Although this is not the place to argue about clinical trial design in SLE, we believe that the failure of the trials is primarily due to the pathogenetic heterogeneity of the disease (36). Heterogeneity implies that each patient or subgroups of patients share a targeted mechanism. Therefore, a distinct subgroup of patients always responds in each trial. It becomes obvious, that there are only a few logical routes to take to success.

Define *a priori* the subgroup of patients in whom the targeted pathway is driving disease and enroll only those. This represents the exercise of personalized or precision medicine which is long overdue in patients with SLE. Alternatively, administer to all patients more than one biologics simultaneously hoping that a larger number of patients will respond. This approach may be stymied by an increased number of side effects.

It is expected that soon a few more biologics will be approved for SLE including the calcineurin inhibitor voclosporin and the IFN blocker anifrolimumab at which point drugs will be prescribed serially to patients with SLE after each one of them fails. This has been the practice in some ways with patients with rheumatoid arthritis and other autoinflammatory diseases.

## Author Contributions

KI, AK, and GT reviewed and edited the manuscript. TK wrote the manuscript. All authors contributed to the article and approved the submitted version.

## Funding

This work was supported by grants from the Leading Initiative for Excellent Young Researchers of the Ministry of Education, Culture, Sports, Science and Technology (to TK, no. 16810055). GT is on the SAB of A2 Therapeutics. Work in the lab of GT has been funded by NIH grants.

## Conflict of Interest

The authors declare that the research was conducted in the absence of any commercial or financial relationships that could be construed as a potential conflict of interest.

## References

[1] Tsokos GC Systemic lupus erythematosus. N Engl J Med (2011) 365:2110–21. 10.1056/NEJMra1100359 22129255

[2] Koga T Ichinose K Kawakami A Tsokos GC The role of IL-17 in systemic lupus erythematosus and its potential as a therapeutic target. Expert Rev Clin Immunol (2019) 15:629–37. 10.1080/1744666X.2019.1593141 30874446

[3] Han JW Zheng HF Cui Y Sun LD Ye DQ Hu Z Genome-wide association study in a Chinese Han population identifies nine new susceptibility loci for systemic lupus erythematosus. Nat Genet (2009) 41:1234–7. 10.1038/ng.472 19838193

[4] Lee HS Kim T Bang SY Na YJ Kim I Kim K Ethnic specificity of lupus-associated loci identified in a genome-wide association study in Korean women. Ann Rheum Dis (2014) 73:1240–5. 10.1136/annrheumdis-2012-202675 23740238

[5] Rullo OJ Tsao BP Recent insights into the genetic basis of systemic lupus erythematosus. Ann Rheum Dis (2013) 72 Suppl 2:ii56–61. 10.1136/annrheumdis-2012-202351 23253915 PMC3780983

[6] Cassia M Alberici F Gallieni M Jayne D Lupus nephritis and B-cell targeting therapy. Expert Rev Clin Immunol (2017) 13:951–62. 10.1080/1744666X.2017.1366855 28800401

[7] Koga T Ichinose K Tsokos GC T cells and IL-17 in lupus nephritis. Clin Immunol (2017) 185:95–9. 10.1016/j.clim.2016.04.010 PMC5074925 27109641

[8] Tsokos GC Autoimmunity and organ damage in systemic lupus erythematosus. Nat Immunol (2020) 21:605–14. 10.1038/s41590-020-0677-6 PMC8135909 32367037

[9] Kolls JK Linden A Interleukin-17 family members and inflammation. Immunity (2004) 21:467–76. 10.1016/j.immuni.2004.08.018 15485625

[10] Waite JC Skokos D Th17 response and inflammatory autoimmune diseases. Int J Inflam (2012) 2012:819467. 10.1155/2012/819467 22229105 PMC3249891

[11] Ouyang W Kolls JK Zheng Y The biological functions of T helper 17 cell effector cytokines in inflammation. Immunity (2008) 28:454–67. 10.1016/j.immuni.2008.03.004 PMC3424508 18400188

[12] Burkett PR Meyer zu Horste G Kuchroo VK Pouring fuel on the fire: Th17 cells, the environment, and autoimmunity. J Clin Invest (2015) 125:2211–9. 10.1172/JCI78085 PMC4497747 25961452

[13] Crispin JC Oukka M Bayliss G Cohen RA Van Beek CA Stillman IE Expanded double negative T cells in patients with systemic lupus erythematosus produce IL-17 and infiltrate the kidneys. J Immunol (2008) 181:8761–6. 10.4049/jimmunol.181.12.8761 PMC2596652 19050297

[14] Sutton CE Mielke LA Mills KH IL-17-producing gammadelta T cells and innate lymphoid cells. Eur J Immunol (2012) 42:2221–31. 10.1002/eji.201242569 22949320

[15] Ruiz de Morales JMG Puig L Dauden E Canete JD Pablos JL Martin AO Critical role of interleukin (IL)-17 in inflammatory and immune disorders: An updated review of the evidence focusing in controversies. Autoimmun Rev (2020) 19:102429. 10.1016/j.autrev.2019.102429 31734402

[16] Apostolidis SA Crispin JC Tsokos GC IL-17-producing T cells in lupus nephritis. Lupus (2011) 20:120–4. 10.1177/0961203310389100 21303828

[17] Moulton VR Tsokos GC T cell signaling abnormalities contribute to aberrant immune cell function and autoimmunity. J Clin Invest (2015) 125:2220–7. 10.1172/JCI78087 PMC4497749 25961450

[18] Wong CK Ho CY Li EK Lam CW Elevation of proinflammatory cytokine (IL-18, IL-17, IL-12) and Th2 cytokine (IL-4) concentrations in patients with systemic lupus erythematosus. Lupus (2000) 9:589–93. 10.1191/096120300678828703 11035433

[19] Wong CK Lit LC Tam LS Li EK Wong PT Lam CW Hyperproduction of IL-23 and IL-17 in patients with systemic lupus erythematosus: implications for Th17-mediated inflammation in auto-immunity. Clin Immunol (2008) 127:385–93. 10.1016/j.clim.2008.01.019 18373953

[20] Vincent FB Northcott M Hoi A Mackay F Morand EF Clinical associations of serum interleukin-17 in systemic lupus erythematosus. Arthritis Res Ther (2013) 15:R97. 10.1186/ar4277 23968496 PMC3979031

[21] Zickert A Amoudruz P Sundstrom Y Ronnelid J Malmstrom V Gunnarsson I IL-17 and IL-23 in lupus nephritis - association to histopathology and response to treatment. BMC Immunol (2015) 16:7. 10.1186/s12865-015-0070-7 25887118 PMC4326189

[22] Schmidt T Paust HJ Krebs CF Turner JE Kaffke A Bennstein SB Function of the Th17/interleukin-17A immune response in murine lupus nephritis. Arthritis Rheumatol (2015) 67:475–87. 10.1002/art.38955 25385550

[23] Lubberts E The IL-23-IL-17 axis in inflammatory arthritis. Nat Rev Rheumatol (2015) 11:562. 10.1038/nrrheum.2015.128 26369609

[24] Cua DJ Sherlock J Chen Y Murphy CA Joyce B Seymour B Interleukin-23 rather than interleukin-12 is the critical cytokine for autoimmune inflammation of the brain. Nature (2003) 421:744–8. 10.1038/nature01355 12610626

[25] Murphy CA Langrish CL Chen Y Blumenschein W McClanahan T Kastelein RA Divergent pro- and antiinflammatory roles for IL-23 and IL-12 in joint autoimmune inflammation. J Exp Med (2003) 198:1951–7. 10.1084/jem.20030896 PMC2194162 14662908

[26] Yen D Cheung J Scheerens H Poulet F McClanahan T McKenzie B IL-23 is essential for T cell-mediated colitis and promotes inflammation via IL-17 and IL-6. J Clin Invest (2006) 116:1310–6. 10.1172/JCI21404 PMC1451201 16670770

[27] Duerr RH Taylor KD Brant SR Rioux JD Silverberg MS Daly MJ A genome-wide association study identifies IL23R as an inflammatory bowel disease gene. Science (2006) 314:1461–3. 10.1126/science.1135245 PMC4410764 17068223

[28] Kyttaris VC Zhang Z Kuchroo VK Oukka M Tsokos GC Cutting edge: IL-23 receptor deficiency prevents the development of lupus nephritis in C57BL/6-lpr/lpr mice. J Immunol (2010) 184:4605–9. 10.4049/jimmunol.0903595 PMC2926666 20308633

[29] Kyttaris VC Kampagianni O Tsokos GC Treatment with anti-interleukin 23 antibody ameliorates disease in lupus-prone mice. BioMed Res Int (2013) 2013:861028. 10.1155/2013/861028 23841097 PMC3690216

[30] Leng RX Pan HF Chen GM Wang C Qin WZ Chen LL IL-23: a promising therapeutic target for systemic lupus erythematosus. Arch Med Res (2010) 41:221–5. 10.1016/j.arcmed.2010.02.011 20682181

[31] Rana A Minz RW Aggarwal R Anand S Pasricha N Singh S Gene expression of cytokines (TNF-alpha, IFN-gamma), serum profiles of IL-17 and IL-23 in paediatric systemic lupus erythematosus. Lupus (2012) 21:1105–12. 10.1177/0961203312451200 22759859

[32] Fuchs TA Abed U Goosmann C Hurwitz R Schulze I Wahn V Novel cell death program leads to neutrophil extracellular traps. J Cell Biol (2007) 176:231–41. 10.1083/jcb.200606027 PMC2063942 17210947

[33] Villanueva E Yalavarthi S Berthier CC Hodgin JB Khandpur R Lin AM Netting neutrophils induce endothelial damage, infiltrate tissues, and expose immunostimulatory molecules in systemic lupus erythematosus. J Immunol (2011) 187:538–52. 10.4049/jimmunol.1100450 PMC3119769 21613614

[34] Pisitkun P Ha HL Wang H Claudio E Tivy CC Zhou H Interleukin-17 cytokines are critical in development of fatal lupus glomerulonephritis. Immunity (2012) 37:1104–15. 10.1016/j.immuni.2012.08.014 PMC3594848 23123062

[35] Lopez P Rodriguez-Carrio J Caminal-Montero L Mozo L Suarez A A pathogenic IFNalpha, BLyS and IL-17 axis in Systemic Lupus Erythematosus patients. Sci Rep (2016) 6:20651. 10.1038/srep20651 26847824 PMC4742957

[36] Tsokos GC Lo MS Costa Reis P Sullivan KE New insights into the immunopathogenesis of systemic lupus erythematosus. Nat Rev Rheumatol (2016) 12:716–30. 10.1038/nrrheum.2016.186 27872476

[37] Wofsy D Ledbetter JA Hendler PL Seaman WE Treatment of murine lupus with monoclonal anti-T cell antibody. J Immunol (1985) 134:852–7. 3871218

[38] Schiffer L Sinha J Wang X Huang W von Gersdorff G Schiffer M Short term administration of costimulatory blockade and cyclophosphamide induces remission of systemic lupus erythematosus nephritis in NZB/W F1 mice by a mechanism downstream of renal immune complex deposition. J Immunol (2003) 171:489–97. 10.4049/jimmunol.171.1.489 12817034

[39] Crispin JC Apostolidis SA Rosetti F Keszei M Wang N Terhorst C Cutting edge: protein phosphatase 2A confers susceptibility to autoimmune disease through an IL-17-dependent mechanism. J Immunol (2012) 188:3567–71. 10.4049/jimmunol.1200143 PMC3324672 22422882

[40] Sunahori K Juang YT Tsokos GC Methylation status of CpG islands flanking a cAMP response element motif on the protein phosphatase 2Ac alpha promoter determines CREB binding and activity. J Immunol (2009) 182:1500–8. 10.4049/jimmunol.182.3.1500 PMC2676107 19155497

[41] Katsiari CG Kyttaris VC Juang YT Tsokos GC Protein phosphatase 2A is a negative regulator of IL-2 production in patients with systemic lupus erythematosus. J Clin Invest (2005) 115:3193–204. 10.1172/JCI24895 PMC1253625 16224536

[42] Apostolidis SA Rodriguez-Rodriguez N Suarez-Fueyo A Dioufa N Ozcan E Crispin JC Phosphatase PP2A is requisite for the function of regulatory T cells. Nat Immunol (2016) 17:556–64. 10.1038/ni.3390 PMC4837024 26974206

[43] Pan W Sharabi A Ferretti A Zhang Y Burbano C Yoshida N PPP2R2D suppresses IL-2 production and Treg function. JCI Insight (2020) 5. 10.1172/jci.insight.138215 PMC7566706 32897879

[44] Thumkeo D Watanabe S Narumiya S Physiological roles of Rho and Rho effectors in mammals. Eur J Cell Biol (2013) 92:303–15. 10.1016/j.ejcb.2013.09.002 24183240

[45] Lee JH Katakai T Hara T Gonda H Sugai M Shimizu A Roles of p-ERM and Rho-ROCK signaling in lymphocyte polarity and uropod formation. J Cell Biol (2004) 167:327–37. 10.1083/jcb.200403091 PMC2172551 15504914

[46] Biswas PS Gupta S Chang E Song L Stirzaker RA Liao JK Phosphorylation of IRF4 by ROCK2 regulates IL-17 and IL-21 production and the development of autoimmunity in mice. J Clin Invest (2010) 120:3280–95. 10.1172/JCI42856 PMC2929726 20697158

[47] Apostolidis SA Rauen T Hedrich CM Tsokos GC Crispin JC Protein phosphatase 2A enables expression of interleukin 17 (IL-17) through chromatin remodeling. J Biol Chem (2013) 288:26775–84. 10.1074/jbc.M113.483743 PMC3772223 23918926

[48] Rozo C Chinenov Y Maharaj RK Gupta S Leuenberger L Kirou KA Targeting the RhoA-ROCK pathway to reverse T-cell dysfunction in SLE. Ann Rheum Dis (2017) 76:740–7. 10.1136/annrheumdis-2016-209850 PMC5839171 28283529

[49] Lippe R Ohl K Varga G Rauen T Crispin JC Juang YT CREMalpha overexpression decreases IL-2 production, induces a T(H)17 phenotype and accelerates autoimmunity. J Mol Cell Biol (2012) 4:121–3. 10.1093/jmcb/mjs004 22355096

[50] Hedrich CM Crispin JC Rauen T Ioannidis C Apostolidis SA Lo MS cAMP response element modulator alpha controls IL2 and IL17A expression during CD4 lineage commitment and subset distribution in lupus. Proc Natl Acad Sci USA (2012) 109:16606–11. 10.1073/pnas.1210129109 PMC3478624 23019580

[51] Rauen T Hedrich CM Juang YT Tenbrock K Tsokos GC cAMP-responsive element modulator (CREM)alpha protein induces interleukin 17A expression and mediates epigenetic alterations at the interleukin-17A gene locus in patients with systemic lupus erythematosus. J Biol Chem (2011) 286:43437–46. 10.1074/jbc.M111.299313 PMC3234851 22025620

[52] Hedrich CM Rauen T Crispin JC Koga T Ioannidis C Zajdel M cAMP-responsive element modulator alpha (CREMalpha) trans-represses the transmembrane glycoprotein CD8 and contributes to the generation of CD3+CD4-CD8- T cells in health and disease. J Biol Chem (2013) 288:31880–7. 10.1074/jbc.M113.508655 PMC3814780 24047902

[53] Hedrich CM Crispin JC Rauen T Ioannidis C Koga T Rodriguez Rodriguez N cAMP responsive element modulator (CREM) alpha mediates chromatin remodeling of CD8 during the generation of CD3+ CD4- CD8- T cells. J Biol Chem (2014) 289:2361–70. 10.1074/jbc.M113.523605 PMC3900979 24297179

[54] Bodor J Feigenbaum L Bodorova J Bare C Reitz MS Jr Gress RE Suppression of T-cell responsiveness by inducible cAMP early repressor (ICER). J Leukoc Biol (2001) 69:1053–9. 11404394

[55] Yoshida N Comte D Mizui M Otomo K Rosetti F Mayadas TN ICER is requisite for Th17 differentiation. Nat Commun (2016) 7:12993. 10.1038/ncomms12993 27680869 PMC5056420

[56] Lin MY Zal T Ch’en IL Gascoigne NR Hedrick SM A pivotal role for the multifunctional calcium/calmodulin-dependent protein kinase II in T cells: from activation to unresponsiveness. J Immunol (2005) 174:5583–92. 10.4049/jimmunol.174.9.5583 15843557

[57] Pan F Means AR Liu JO Calmodulin-dependent protein kinase IV regulates nuclear export of Cabin1 during T-cell activation. EMBO J (2005) 24:2104–13. 10.1038/sj.emboj.7600685 PMC1150881 15902271

[58] McGargill MA Sharp LL Bui JD Hedrick SM Calbo S Active Ca2+/calmodulin-dependent protein kinase II gamma B impairs positive selection of T cells by modulating TCR signaling. J Immunol (2005) 175:656–64. 10.4049/jimmunol.175.2.656 16002660

[59] Raman V Blaeser F Ho N Engle DL Williams CB Chatila TA Requirement for Ca2+/calmodulin-dependent kinase type IV/Gr in setting the thymocyte selection threshold. J Immunol (2001) 167:6270–8. 10.4049/jimmunol.167.11.6270 11714790

[60] Racioppi L Means AR Calcium/calmodulin-dependent kinase IV in immune and inflammatory responses: novel routes for an ancient traveller. Trends Immunol (2008) 29:600–7. 10.1016/j.it.2008.08.005 18930438

[61] Juang YT Wang Y Solomou EE Li Y Mawrin C Tenbrock K Systemic lupus erythematosus serum IgG increases CREM binding to the IL-2 promoter and suppresses IL-2 production through CaMKIV. J Clin Invest (2005) 115:996–1005. 10.1172/JCI22854 15841182 PMC1070410

[62] Koga T Ichinose K Mizui M Crispin JC Tsokos GC Calcium/Calmodulin-Dependent Protein Kinase IV Suppresses IL-2 Production and Regulatory T Cell Activity in Lupus. J Immunol (2012) 189:3490–6. 10.4049/jimmunol.1201785 PMC3448834 22942433

[63] Koga T Hedrich CM Mizui M Yoshida N Otomo K Lieberman LA CaMK4-dependent activation of AKT/mTOR and CREM-alpha underlies autoimmunity-associated Th17 imbalance. J Clin Invest (2014) 124:2234–45. 10.1172/JCI73411 PMC4001553 24667640

[64] Ichinose K Rauen T Juang YT Kis-Toth K Mizui M Koga T Cutting edge: Calcium/Calmodulin-dependent protein kinase type IV is essential for mesangial cell proliferation and lupus nephritis. J Immunol (2011) 187:5500–4. 10.4049/jimmunol.1102357 PMC3221799 22031763

[65] Koga T Mizui M Yoshida N Otomo K Lieberman LA Crispin JC KN-93, an inhibitor of calcium/calmodulin-dependent protein kinase IV, promotes generation and function of Foxp3(+) regulatory T cells in MRL/lpr mice. Autoimmunity (2014) 47:445–50. 10.3109/08916934.2014.915954 PMC4341833 24829059

[66] Koga T Otomo K Mizui M Yoshida N Umeda M Ichinose K CaMK4 facilitates the recruitment of IL-17-producing cells to target organs through the CCR6/CCL20 axis in Th17-driven inflammatory diseases. Arthritis Rheumatol (2016). 10.1002/art.39665 PMC4963275 26945541

[67] Koga T Sato T Furukawa K Morimoto S Endo Y Umeda M Calcium/calmodulin-dependent protein kinase 4 promotes GLUT1-dependent glycolysis in systemic lupus erythematosus. Arthritis Rheumatol (2018). 10.1002/art.40785 30462889

[68] Otomo K Koga T Mizui M Yoshida N Kriegel C Bickerton S Cutting Edge: Nanogel-Based Delivery of an Inhibitor of CaMK4 to CD4+ T Cells Suppresses Experimental Autoimmune Encephalomyelitis and Lupus-like Disease in Mice. J Immunol (2015) 195:5533–7. 10.4049/jimmunol.1501603 PMC4670795 26561550

[69] Perl A Review: Metabolic Control of Immune System Activation in Rheumatic Diseases. Arthritis Rheumatol (2017) 69:2259–70. 10.1002/art.40223 PMC5711528 28841779

[70] Bhaskar PT Hay N The two TORCs and Akt. Dev Cell (2007) 12:487–502. 10.1016/j.devcel.2007.03.020 17419990

[71] Caza TN Fernandez DR Talaber G Oaks Z Haas M Madaio MP HRES-1/Rab4-mediated depletion of Drp1 impairs mitochondrial homeostasis and represents a target for treatment in SLE. Ann Rheum Dis (2014) 73:1888–97. 10.1136/annrheumdis-2013-203794 PMC4047212 23897774

[72] Powell JD Delgoffe GM The mammalian target of rapamycin: linking T cell differentiation, function, and metabolism. Immunity (2010) 33:301–11. 10.1016/j.immuni.2010.09.002 PMC2962404 20870173

[73] Gentry EG Henderson BW Arrant AE Gearing M Feng Y Riddle NC Rho Kinase Inhibition as a Therapeutic for Progressive Supranuclear Palsy and Corticobasal Degeneration. J Neurosci (2016) 36:1316–23. 10.1523/JNEUROSCI.2336-15.2016 PMC4728727 26818518

[74] Katsuyama T Li H Comte D Tsokos GC Moulton VR Splicing factor SRSF1 controls T cell hyperactivity and systemic autoimmunity. J Clin Invest (2019) 129:5411–23. 10.1172/JCI127949 PMC6877308 31487268

[75] Lai ZW Borsuk R Shadakshari A Yu J Dawood M Garcia R Mechanistic target of rapamycin activation triggers IL-4 production and necrotic death of double-negative T cells in patients with systemic lupus erythematosus. J Immunol (2013) 191:2236–46. 10.4049/jimmunol.1301005 PMC3777662 23913957

[76] Kato H Perl A Mechanistic target of rapamycin complex 1 expands Th17 and IL-4+ CD4-CD8- double-negative T cells and contracts regulatory T cells in systemic lupus erythematosus. J Immunol (2014) 192:4134–44. 10.4049/jimmunol.1301859 PMC3995867 24683191

[77] Kono M Yoshida N Maeda K Tsokos GC Transcriptional factor ICER promotes glutaminolysis and the generation of Th17 cells. Proc Natl Acad Sci U S A (2018) 115:2478–83. 10.1073/pnas.1714717115 PMC5877961 29463741

[78] Kono M Yoshida N Maeda K Suarez-Fueyo A Kyttaris VC Tsokos GC Glutaminase 1 Inhibition Reduces Glycolysis and Ameliorates Lupus-like Disease in MRL/lpr Mice and Experimental Autoimmune Encephalomyelitis. Arthritis Rheumatol (2019) 71:1869–78. 10.1002/art.41019 PMC6817384 31233276

[79] Kato H Perl A Blockade of Treg Cell Differentiation and Function by the Interleukin-21-Mechanistic Target of Rapamycin Axis Via Suppression of Autophagy in Patients With Systemic Lupus Erythematosus. Arthritis Rheumatol (2018) 70:427–38. 10.1002/art.40380 PMC5826851 29161463

[80] Huang N Perl A Metabolism as a Target for Modulation in Autoimmune Diseases. Trends Immunol (2018) 39:562–76. 10.1016/j.it.2018.04.006 29739666

[81] Lai ZW Kelly R Winans T Marchena I Shadakshari A Yu J Sirolimus in patients with clinically active systemic lupus erythematosus resistant to, or intolerant of, conventional medications: a single-arm, open-label, phase 1/2 trial. Lancet (2018) 391:1186–96. 10.1016/S0140-6736(18)30485-9 PMC5891154 29551338

[82] Langley RG Elewski BE Lebwohl M Reich K Griffiths CE Papp K Secukinumab in plaque psoriasis–results of two phase 3 trials. N Engl J Med (2014) 371:326–38. 10.1056/NEJMoa1314258 25007392

[83] Griffiths CE Reich K Lebwohl M van de Kerkhof P Paul C Menter A Comparison of ixekizumab with etanercept or placebo in moderate-to-severe psoriasis (UNCOVER-2 and UNCOVER-3): results from two phase 3 randomised trials. Lancet (2015) 386:541–51. 10.1016/S0140-6736(15)60125-8 26072109

[84] Lebwohl M Strober B Menter A Gordon K Weglowska J Puig L Phase 3 Studies Comparing Brodalumab with Ustekinumab in Psoriasis. N Engl J Med (2015) 373:1318–28. 10.1056/NEJMoa1503824 26422722

[85] McInnes IB Mease PJ Kirkham B Kavanaugh A Ritchlin CT Rahman P Secukinumab, a human anti-interleukin-17A monoclonal antibody, in patients with psoriatic arthritis (FUTURE 2): a randomised, double-blind, placebo-controlled, phase 3 trial. Lancet (2015) 386:1137–46. 10.1016/S0140-6736(15)61134-5 26135703

[86] Mease PJ Genovese MC Greenwald MW Ritchlin CT Beaulieu AD Deodhar A Brodalumab, an anti-IL17RA monoclonal antibody, in psoriatic arthritis. N Engl J Med (2014) 370:2295–306. 10.1056/NEJMoa1315231 24918373

[87] Baeten D Sieper J Braun J Baraliakos X Dougados M Emery P Secukinumab, an Interleukin-17A Inhibitor, in Ankylosing Spondylitis. N Engl J Med (2015) 373:2534–48. 10.1056/NEJMoa1505066 26699169

[88] Pavelka K Kivitz A Dokoupilova E Blanco R Maradiaga M Tahir H Efficacy, safety, and tolerability of secukinumab in patients with active ankylosing spondylitis: a randomized, double-blind phase 3 study, MEASURE 3. Arthritis Res Ther (2017) 19:285. 10.1186/s13075-017-1490-y 29273067 PMC5741872

[89] Satoh Y Nakano K Yoshinari H Nakayamada S Iwata S Kubo S A case of refractory lupus nephritis complicated by psoriasis vulgaris that was controlled with secukinumab. Lupus (2018) 27:1202–6. 10.1177/0961203318762598 29523055

[90] De Souza A Ali-Shaw T Strober BE Franks AG Jr Successful treatment of subacute lupus erythematosus with ustekinumab. Arch Dermatol (2011) 147:896–8. 10.1001/archdermatol.2011.185 21844448

[91] Leonardi CL Kimball AB Papp KA Efficacy and safety of ustekinumab, a human interleukin-12/23 monoclonal antibody, in patients with psoriasis: 76-week results from a randomised, double-blind, placebo-controlled trial (PHOENIX 1). Lancet (2008) 371:1665–74. 10.1016/S0140-6736(08)60725-4 18486739

[92] McInnes IB Kavanaugh A Gottlieb AB Efficacy and safety of ustekinumab in patients with active psoriatic arthritis: 1 year results of the phase 3, multicentre, double-blind, placebo-controlled PSUMMIT 1 trial. Lancet (2013) 382:780–9. 10.1016/S0140-6736(13)60594-2 23769296

[93] van Vollenhoven RF Hahn BH Tsokos GC Wagner CL Lipsky P Touma Z Efficacy and safety of ustekinumab, an IL-12 and IL-23 inhibitor, in patients with active systemic lupus erythematosus: results of a multicentre, double-blind, phase 2, randomised, controlled study. Lancet (2018) 392:1330–9. 10.1016/S0140-6736(18)32167-6 30249507

[94] Cesaroni M Seridi L Loza MJ Schreiter J Sweet K Franks C Response to ustekinumab treatment in patients with systemic lupus erythematosus is linked to suppression of serum interferon gamma levels. Arthritis Rheumatol (2020). 10.1002/art.41547 PMC7986128 33010188

